# Supplementary material for: Blood Cell-Derived Inflammatory Indices in Diabetic Macular Edema: Clinical Significance and Prognostic Relevance
Source: Biomedicines. 2025 Dec 4;13(12):2979. doi: 10.3390/biomedicines13122979 (PMC12730579; doi:10.3390/biomedicines13122979)
Supplement: Supplementary file 1 [file biomedicines-13-02979-s001.zip › Supplementary Information S1.pdf]

Supplementary Information S1. Immune Mechanisms and Pathways of Peripheral Blood-Derived Inflammatory Indices in Diabetic Macular Edema

Index	Cellular composition	Immune axis (Innate/Adaptive/ Mixed)	Mechanistic pathways involved	References
NLR	Neutrophils / Lymphocytes	Mainly innate ↑ / adaptive ↓	Neutrophil-mediated proinflammatory activity (ROS, NETs); lymphocyte immunoregulatory deficiency → BRB disruption and retinal edema	[1,2]
PLR	Platelets / Lymphocytes	Mixed (platelet-mediated + adaptive)	Platelet activation, platelet–neutrophil/monocyte aggregates, promotion of angiogenesis (e.g., VEGF-related) → increased microvascular permeability	[2]
SII	(Neutrophils × Platelets) / Lymphocytes	Mixed (innate + platelet + adaptive)	Reflects combined neutrophil and platelet proinflammatory activity relative to lymphocytes → systemic inflammatory burden ↑ → risk of DME and specific subtypes (e.g., SRD)	[3]
MLR	Monocytes / Lymphocytes	Mixed (innate + adaptive)	Monocyte/macrophage axis activation → secretion of IL-6, TNF-α → vascular permeability and fibrosis	[4]
PIV	(Platelets × Neutrophils × Monocytes) / Lymphocytes	Mixed (platelet + innate + adaptive)	Similar to SII but includes broader cell spectrum, reflecting overall immune/inflammatory status	[5]

* NLR: neutrophil-to-lymphocyte ratio; PLR: platelet-to-lymphocyte ratio; SII: systemic immune-inflammation index; MLR: monocyte-to-lymphocyte ratio; PIV: pan-immune-inflammation value; ROS: reactive oxygen species; NETs: neutrophil extracellular traps; BRB: blood-retinal barrier; VEGF: vascular endothelial growth factor; DME: diabetic macular edema; SRD: serous retinal detachment; IL-6: interleukin-6; TNF-α: tumor necrosis factor-alpha.

References

1. Hu, Y.; Cheng, Y.; Xu, X.; Yang, B.; Mei, F.; Zhou, Q.; Yan, L.; Wang, J.; Wu, X. Pretreatment neutrophil-to-lymphocyte ratio predicts prognosis in patients with diabetic macular edema treated with ranibizumab. *BMC Ophthalmol* **2019**, *19*, 194, doi:10.1186/s12886-019-1200-4.
2. Gu, W.; Wang, M.; Li, Z.; Xu, T. The association between peripheral blood inflammatory markers and anti-VEGF treatment response in patients with type 2 diabetic macular edema. *Front Med (Lausanne)* **2025**, *12*, 1653753, doi:10.3389/fmed.2025.1653753.
3. Elbeyli, A.; Kurtul, B.E.; Ozcan, S.C.; Ozarslan Ozcan, D. The diagnostic value of systemic immune-inflammation index in diabetic macular oedema. *Clin Exp Optom* **2022**, *105*, 831-835, doi:10.1080/08164622.2021.1994337.
4. He, X.; Qi, S.; Zhang, X.; Pan, J. The relationship between the neutrophil-to-lymphocyte ratio and diabetic retinopathy in adults from the United States: results from the National Health and nutrition examination survey. *BMC Ophthalmol* **2022**, *22*, 346, doi:10.1186/s12886-022-02571-z.
5. Candan, O.; Orman, G.; Ünlü, N.; Ozkan, G. Diagnostic efficiency of pan-immune-inflammation value to predict diabetic macular edema and its relationship with OCT-based biomarkers of inflammation. *Int Ophthalmol* **2025**, *45*, 365, doi:10.1007/s10792-025-03733-w.
