# Supplementary material for: Blood Cell-Derived Inflammatory Indices in Diabetic Macular Edema: Clinical Significance and Prognostic Relevance
Source: Biomedicines. 2025 Dec 4;13(12):2979. doi: 10.3390/biomedicines13122979 (PMC12730579; doi:10.3390/biomedicines13122979)
Supplement: Supplementary file 1 [file biomedicines-13-02979-s001.zip › Supplementary information S2.pdf]

Supplementary Information S2. Evidence grading and clinical classification of blood cell-derived inflammatory indices in diabetic macular edema

1. Literature Search

A systematic literature search was conducted in three electronic databases (PubMed, Embase, and Web of Science) from inception to July 2025. The search strategy combined Medical Subject Headings (MeSH) terms and free-text keywords related to diabetic macular edema (DME), blood cell-derived inflammatory indices, optical coherence tomography (OCT), and anti-vascular endothelial growth factor (VEGF). The search terms included ("diabetic macular edema" OR "DME") AND ("neutrophil-to-lymphocyte ratio" OR "NLR" OR "platelet-to-lymphocyte ratio" OR "PLR" OR "systemic immune-inflammation index" OR "SII" OR "monocyte-to-high-density lipoprotein cholesterol ratio" OR "MHR" OR "monocyte-to-lymphocyte ratio" OR "MLR" OR "pan-immune-inflammation value" OR "PIV" OR "mean platelet volume" OR "MPV" OR "platelet distribution width" OR "PDW") OR ("diffuse retinal thickening" OR "DRT" OR "cystoid macular edema" OR "CME" OR "serous retinal detachment" OR "SRD" OR "anti-vascular endothelial growth factor" OR "anti-VEGF"). Only studies involving human participants were included. No language restrictions were applied.

Titles and abstracts were independently screened by two authors (Chiyu Lin and Weiqing Ye), with discrepancies resolved through consensus. Full-text articles were reviewed for potentially eligible studies. This narrative review format was selected due to substantial heterogeneity in study designs, populations, and outcome measures, which precluded the use of a quantitative meta-analysis. An initial literature search identified approximately 21 studies for this review. However, only 13 were ultimately suitable for evidence grading and clinical classification of blood cell-derived inflammatory indices in diabetic macular edema due to incomplete data reporting.

2. Justification for marker-level evidence grading

The evidence base comprised exclusively of observational studies—prospective and retrospective cohorts, along with case–control designs—with no randomized trials available. Substantial heterogeneity characterized the literature, spanning patient selection criteria, comparator definitions (healthy controls versus diabetic eyes without DME), OCT-based DME subtypes, and anti-VEGF response outcomes. Furthermore, many studies reported data in formats that precluded formal meta-analysis, lacking effect estimates with 95% confidence intervals.

In these circumstances, AHRQ Evidence-based Practice Center (EPC) guidance and the GRADE Working Group both advocate grading the certainty of an evidence body based on core domains—risk of bias, consistency, directness, and precision—rather than forcing unstable quantitative synthesis. GRADE posits that evidence from observational studies begins at low certainty, with upgrades reserved for specific scenarios (e.g., very large effect sizes or clear dose–response relationships). Our data met few, if any, of these upgrade criteria. A full outcome-by-outcome GRADE assessment would therefore have yielded uniformly "low" or "very low" certainty ratings, obscuring meaningful distinctions between indices.

We therefore implemented a pragmatic, marker-level framework that retains the foundational principles of AHRQ and GRADE while accommodating the heterogeneity of our observational evidence. Rather than grading outcomes individually, we aggregated all studies for each marker into a single evidence body, to which we assigned an overall grade and clinical category.

3. Data preparation and extraction

We compiled the raw dataset (Supplementary Information 3) from all eligible studies reporting peripheral inflammatory indices in diabetic macular edema. For each study–marker combination (NLR, PLR, SII, MPV, MLR, MHR), we extracted bibliographic details (author, year); clinical context (DME versus non-DME discrimination, OCT-based subtyping, anti-VEGF response prediction); study design (prospective versus retrospective); group descriptors and sample sizes; means and standard deviations; and ROC metrics (AUC, sensitivity, specificity), where available.

When a single study reported multiple comparisons for the same marker (e.g., DME versus non-DME plus OCT subtype analyses), we treated these as distinct clinical contexts within a single study–marker unit. To approximate the effective sample size per study for each marker, we calculated the maximum total N (group 1 + group 2) across all relevant comparisons and used this as the study's representative sample size for that marker. The distribution across studies is summarized as median and range (Table 3).

4. Streamlined evaluation of core AHRQ/GRADE domains

Given reporting limitations, we applied a qualitative synthesis of the four AHRQ/GRADE domains rather than a formal study-level risk-of-bias assessment with QUADAS-2 or ROBINS-I.

4.1 Risk of bias (study limitations)

We evaluated study design (prospective versus retrospective), comparator group adequacy, and the extent of confounder adjustment as reported in each study. Prospective studies with explicit inclusion criteria and confounder adjustment were deemed lower risk than small, single-center retrospective analyses lacking such controls. Given that our evidence base was purely observational, we treated the overall risk of bias as at least moderate for all markers, consistent with GRADE's default position for observational data. In addition, we generated a simplified study-level judgment (moderate versus high) for each article based on study design and whether multivariable adjustment for major confounders was reported; these qualitative ratings are summarized in Supplementary Information 4 and should not be interpreted as a formal QUADAS-2 or ROBINS-I assessment.

4.2 Consistency

We assessed whether studies demonstrated concordant effect directions (e.g., uniformly elevated NLR in DME versus controls) and whether AUC values clustered within a comparable range. Indices with numerous studies showing largely aligned findings across contexts were judged more consistent than those with sparse or contradictory data.

4.3 Directness

All studies directly examined associations between peripheral inflammatory indices and either DME presence, OCT-defined subtypes, or anti-VEGF treatment response in diabetic patients. No surrogate populations or indirect comparisons were used, so the overall directness was considered good.

4.4 Precision

Because many studies lacked odds ratios or hazard ratios with confidence intervals, we used total sample size and AUC variability as surrogate indicators, interpreting larger studies with tighter AUC ranges as more precise than small, highly variable ones.

5. Evidence tiers at the marker level

Building on the AHRQ EPC framework—which integrates risk of bias, consistency, directness, and precision into overall ratings of high, moderate, low, or insufficient—we established three pragmatic evidence tiers:

- Moderate (Grade B): ≥8 independent observational studies spanning ≥2 clinical contexts (diagnostic discrimination, subtyping, treatment prediction); effects directionally consistent without major contradictions.
- Limited (Grade C): 3–7 observational studies restricted to 1–2 contexts and/or with some variability/uncertainty.
- Very limited: ≤2 studies, typically single-center retrospective; hypothesis-generating only.

These numerical thresholds and clinical category labels are not dictated by any single guideline; they were pre-specified by the authors as pragmatic rules for this review. Using this schema, NLR, PLR, and SII qualified as moderate (Grade B); MPV and MLR as limited (Grade C); and MHR as very limited (Table 3).

6. Clinical classification of indices

To enhance interpretability, we cross-tabulated evidence tiers with discriminative performance (AUC) from studies reporting ROC analyses. Following established benchmarks (0.5–0.7: poor; 0.7–0.8: acceptable; 0.8–0.9: excellent; >0.9: outstanding), we defined the following:

- Core indices (NLR, PLR, SII): Moderate evidence (Grade B), evaluated in ≥2 contexts, with ≥1 study showing acceptable AUC (≥0.7).
- Secondary indices (MPV, MLR): Limited evidence (Grade C), fewer studies/contexts, AUC values mostly 0.55–0.75.
- Exploratory indices (MHR): Sparse evidence, single-center retrospective, no established clinical role.

7. Acknowledged limitations

We recognize that this framework represents a deliberate simplification that deviates from full GRADE assessment in several respects: we did not perform formal, item-by-item risk-of-bias evaluation for each study; we graded markers rather than individual outcomes; and we assessed precision qualitatively rather than via meta-analytic confidence intervals. These choices reflect the heterogeneity and reporting gaps in the current literature and the narrative

scope of this review. Our grades should thus be interpreted as pragmatic, marker-level syntheses intended to differentiate relatively well-studied markers from exploratory ones, rather than as definitive, outcome-specific GRADE assessments.
