# Supplementary material for: Blood Cell-Derived Inflammatory Indices in Diabetic Macular Edema: Clinical Significance and Prognostic Relevance
Source: Biomedicines. 2025 Dec 4;13(12):2979. doi: 10.3390/biomedicines13122979 (PMC12730579; doi:10.3390/biomedicines13122979)
Supplement: Supplementary file 1 [file biomedicines-13-02979-s001.zip › Supplementary information S4.pdf]

Supplementary Information S4. Simplified risk-of-bias assessment for included observational studies

Author, year	Study design	Control group/comparison	Adjustment for confounders	Overall risk of bias*
Akl et al. 2024	Retrospective	DME vs. non-DME controls	No	High
Candan et al. 2025	Retrospective	DME vs. non-DME controls	Yes	Moderate
Chen et al. 2025	Retrospective	Responders vs. non-responders	Yes	Moderate
Elbeyli et al. 2022	Prospective	DME vs. non-DME controls; OCT-based DME subtypes (SMD vs. non-SMD)	No	Moderate
Ergin et al. 2025	Retrospective	Responders vs. non-responders	No	High
Ilhan et al. 2020	Prospective	DME vs. non-DME controls	No	Moderate
Katić et al. 2024	Prospective	Responders vs. non-responders	Yes	Moderate
Li et al. 2018	Retrospective	DME vs. non-DME controls	Yes	Moderate
Liao et al. 2024	Retrospective	DME vs. non-DME controls; OCT-based DME subtypes (CME vs. non-CME)	No	High
Sun et al. 2025	Retrospective	DME vs. non-DME controls; OCT-based DME subtypes (SMD vs. non-SMD)	Yes	Moderate
Yalinbas Yeter et al. 2022	Retrospective	DME vs. non-DME controls	No	High
Zhu et al. 2022	Retrospective	DME vs. non-DME controls	No	High
Özata Gündoğdu et al. 2022	Retrospective	DME vs. non-DME controls; OCT-based DME subtypes (SMD vs. non-SMD)	No	High

* Overall risk of bias was judged qualitatively (low/moderate/high) for each study, considering the study design (prospective vs. retrospective), appropriateness of the control group or comparison (population selection), and adjustment for key confounders. Because all studies were observational, none were considered to have a truly low risk of bias; ratings were restricted to moderate or high. In this simplified scheme, prospective studies and retrospective studies with multivariable adjustment for major confounders (e.g., age, duration of diabetes, systemic risk factors) were rated as moderate risk of bias, whereas unadjusted retrospective studies were rated as high risk of bias. This approach follows the general risk-of-bias domains used in AHRQ and GRADE frameworks but does not rely on a formal tool such as QUADAS-2 or ROBINS-I and is acknowledged as a limitation of the review.
